# Long non-coding RNA LOC366613 alleviates the cerebral ischemic injury via regulating the miR-532-5p/phosphatase and tensin homolog axis

**DOI:** 10.1080/21655979.2021.1930966

**Published:** 2021-07-12

**Authors:** Zhenze Lu, Ling Li, Lei Wei, Jifu Cai, Jun Wu

**Affiliations:** a Guangzhou Medical University Graduate School; b Neurology, Department of Medicine, The University of Hong Kong-Shenzhen Hospital; c Department of Neurology, The Third Affiliated Hospital of Sun Yat-Sen University; d Department of Neurology, Peking University Shenzhen Hospital

**Keywords:** LOC366613, miR-532-5p, cerebral ischemia injury, phosphatase and tensin homolog, apoptosis

## Abstract

Cerebral infarction (CI) has become a leading cause of death in China. Long non-coding RNAs (lncRNAs) are intensively involved in the progression of CI. Here, we aimed to investigate the effects of lncRNA LOC366613 (LOC366613) on cerebral I/R injury, as well as its possible mechanism. Transient middle cerebral artery occlusion (MCAO) was used to establish a mouse model of cerebral I/R, and the PC12 cell line was used to establish an *in vitro* oxygen-glucose deprivation (OGD) injury model. The MTT assay was used to determine cell viability, and qRT-PCR was used to determine RNA levels. Western blotting was conducted to detect protein expression levels. The TUNEL assay and flow cytometry were used to measure cell apoptosis, and 2,3,5-triphenyltetrazolium chloride (TTC) was used to determine cerebral infarct volume. Finally, RNA pull-down and luciferase activity assays were used to examine interactions between miR-532-5p and LOC366613, as well as between miR-532-5p and phosphatase and tensin homolog (PTEN). LOC366613 was overexpressed in patients with cerebral I/R injury. In PC12 cells, knockdown of LOC366613 reduced the apoptosis rate and lactic acid dehydrogenase (LDH) expression, while increasing cell viability. Moreover, miR-532-5p was shown to be a target of LOC366613, as predicted. Downregulation of miR-532-5p reversed the effects of LOC366613 knockdown on PC12 cell apoptosis, LDH release, and cell viability. Finally, *PTEN* was verified as a target of miR-532-5p. LOC366613 participates in cerebral I/R injury by regulating the miR-532-5p/*PTEN* axis, potentially providing a new CI treatment target.

## GRAPHICAL ABSTRACT

**Figure:**
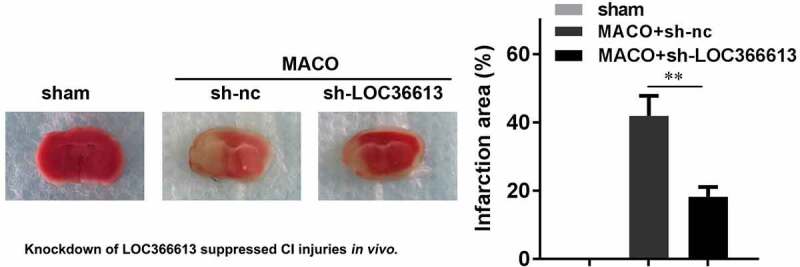

## Introduction

Cerebral infarction (CI) is one of the leading causes of death in China [1,2]. It is characterized by high mortality and disability rates, and over 80% of instances are caused by cerebral ischemia and hypoxic necrosis [3]. Thus far, the most effective way to reduce cerebral ischemic injury is to restore blood perfusion to affected tissues. However, excessive quantities of free radicals may be produced during the recovery from blood reperfusion therapy, resulting in ischemia/reperfusion (I/R) injury [4]. Dysfunction of neuronal cell and inflammatory responses play essential roles in CI [5,6]. Therefore, understanding neuronal apoptosis and inflammation mechanisms may lead to additional strategies for cerebral injury prevention, as well as adjuvant therapies.

lncRNAs are transcribed RNA molecules with a length of more than 200 nucleotides that do not bind to proteins [7]. Recent studies have shown that lncRNAs regulate important biological processes, including cell proliferation, survival, apoptosis, and differentiation [8–10]. They have also been found to be associated with cerebral I/R injury [11–13]. For example, knockdown of lnc-Gm11974 reduces cerebral I/R injury by alleviating apoptosis [14]. However, the roles that lncRNAs play in cerebral I/R injury still require further exploration.

Previous studies have suggested that lncRNAs regulate cerebral I/R injury by acting as sponges for miRNAs [15]. Dysregulated miRNAs play a crucial role in the occurrence of various diseases, such as cancer, type 2 diabetes mellitus, and cerebral I/R injury, as well as in protection against it [16–19]. Liang *et al*. found that miRNA-320 promotes cerebral I/R injury in mice by inhibiting IGF-1 expression [20]. Similarly, miRNA-128-3p promotes cell proliferation by regulating Nrf2, which inhibits cell apoptosis and alleviates oxidative stress [21]. miRNA-128-3p also has a protective effect against cerebral I/R injury. Additionally, downregulation of *BMF* and *BCL2L13* via miR-874-3p overexpression reduces cerebral I/R injury [22]. The phosphatase and tensin homolog (PTEN) gene, which codes for 403 amino acids, exhibits phosphatase activity and dephosphorylates lipid substrates and proteins [23]. miR-532-5p exerts neuron-protective role and its downregulation confers cerebral I/R oxidative stress injury [24,25]. However, the potential roles of miR-532-5p in CI injuries has not been fully elucidated.

PTEN plays a key role in regulating cell proliferation, apoptosis, and migration, as well as in I/R-induced injuries in the kidneys and I/R injury in cardiomyocytes [26]. Conversely, PTEN overexpression inhibits myocardial cell viability, promotes myocardial cell apoptosis, and aggravates myocardial I/R injury [27]. However, more research is needed to understand the regulatory role that the miR-532-5p/PTEN axis plays in cerebral I/R injuries.

In this study, we reported that LOC366613 was upregulated in an oxygen-glucose deprivation (OGD)-induced PC12 cell line and mouse I/R model. Moreover, LOC366613 functioned as a ceRNA and sponged miR-532-5p to regulate the expression of PTEN, which further promoted the progression of CI. These data indicate that LOC366613 could be a potential therapeutic target and biomarker for monitoring human cerebral I/R injury.

## Materials and methods

### Animal model

A total of seventy-five 8-week-old male C57BL/6 J mice (20 ~ 25 g) were purchased from the Experimental Animal Center of Guangdong Province and housed in an SPF environment without specific pathogens (18 ~ 22°C, humidity 65%, alternating light and dark periods of 12 h) and with free access to food and water. Mice were randomly divided into three groups: sham group (n = 10), transient middle cerebral artery occlusion (MCAO) +sh-NC group (n = 10), and MCAO + sh-LOC366613 group (n = 10). All mice were anesthetized with 5% chloral hydrate mixed with oxygen and nitrogen. Mice in sham group underwent similar surgery procedures without inserting suture. The mice before MCAO surgery was intracerebroventricularly injected with 0.5 mg/kg of sh-LOC366613 and sh-NC. A 2 cm length of a 7–0 monofilament suture was gently inserted from the external carotid artery up to the internal carotid artery to occlude the middle cerebral artery until regional cerebral blood flow was reduced to <16% of baseline. After 1 h of MCAO surgery, blood flow was restored by removing the suture. The mice were allowed to recover for 1–7 day. Subsequently, mice were euthanized with pentobarbital sodium (160 mg/kg, IP), a standard and acceptable euthanasia method, and decapitated for brain dissection.

This study was supervised by the Animal Care Board of Peking University Shenzhen Hospital.

### Cerebral infarct volume assay

The brains were cut into six coronal sections (2 mm thick) and stained with 2% TTC (Sigma, St. Louis, MO, USA) at 37°C for 15 min.

### LDH activity assay

Forty-eight hours after transfection, the LDH Activity Kit (Beyotime Institute of Biotechnology, Shanghai) was used to detect the LDH activity of each cell group, according to the manufacturer’s protocol. A microplate reader (Benchmark Plus, Bio-Rad, Hercules, USA) was used to measure absorbance at a wavelength of 450 nm.

### TUNEL assay

PC12 cells were fixed with 4% paraformaldehyde at room temperature before incubation in a permeabilization solution on ice for 3 min. After the TUNEL reaction mixture was added to the cells, the cells were incubated at 37°C for 1 h in dark and humid conditions (5% CO_2_). Finally, the cells were stained with 1 μg/mL 4′,6-diamidino-2-phenylindole (DAPI, Thermo Fisher Scientific, Waltham, USA) for 15 min. Phosphate buffered saline (PBS) was used to rinse the samples, and a fluorescence microscope (Olympus, Tokyo, Japan) was used to observe the images. The number of TUNEL-positive nuclei in the samples was calculated, along with the percentage of apoptotic cells present.

TUNEL testing was performed using a TUNEL FITC Apoptosis Detection kit (Roche Applied Science, Mannheim, Germany). The brain tissues were washed, dehydrated, embedded in paraffin, and cut into 4 μm-thick sections, with the experimental procedure performed according to manufacturer instructions. Next, the sections were incubated with a methanol solution containing 0.2% H_2_O_2_ for 0.5 h to block endogenous peroxidase activity. The number of TUNEL-positive nuclei and the percentage of apoptotic cells present in the tissues were calculated.

### Immunofluorescence assay

The sections were immersed in a 30% sucrose solution in PBS overnight and permeabilized. PBS with 5% goat serum was used to block nonspecific binding for 1 h at room temperature. Then the slides were incubated first with primary antibodies against IBA1 and PTEN, and then with a relative secondary antibody (1:50 dilution; Dianova). Then, their nuclei were stained with DAPI (Thermo Fisher Scientific). After staining, the slides were washed twice with PBS and covered with DABCO (Sigma-Aldrich, St. Louis, USA). Finally, images were observed using a fluorescence microscope.

### Cell culture and transfection

PC12 cells were purchased from Shanghai Institutes of Cell Biological Sciences (Shanghai, China), and cultured in Dulbecco’s Modified Eagle Medium (DMEM, Gibco, USA) with 10% fetal bovine serum at 37°C with 5% CO_2_. Cells, at 2 ~ 3 phases, were used in the following experiment.

Cells incubated with glucose-free DMEM containing 95% N2, 1% O_2_, and 4% CO_2_ at 37°C for 6 h.

The miR-532-5p mimic, miR-532-5p inhibitor, LOC366613 siRNA, and LOC366613 shRNA were synthesized by GenePharma (Shanghai, China). Full-length LOC366613 segments were cloned into pcDNA3.1 vectors. Lipofectamine 2000 (Invitrogen, USA) was used for transfection according to manufacturer’s instructions.

Sh-LOC366613 was introduced into pLVX-Puro vector. Lentivirus particles were generated by HEK293T cells containing sh-LOC366613 using Lipofectamine 2000. Then cell supernatants were collected. Afterward, PC12 cells were transfected with lentivirus particles containing the pLVX-Puro vector bearing human sh-LOC366613.

### MTT assay

MTT assay were used to evaluate the viability of PC12 cells. PC12 cells (10^4^ cells/cm^2^) were seed into 96-well plates. Next, each well was added 16 µL/well MTT solution (5 mg/mL, Sigma) and incubated for 4 h at 37°C. Then cell supernatants were removed and added with 150 µl of DMSO to allow the crystals to fully melt. Afterward, the mixture were dissolved in 100 µl of dimethyl sulfoxide. Cell viability was determined by a micro-plate reader (Bio-Tek, Winooski, USA) at absorbance values of 570 nm.

### Apoptosis assay

An Annexin V-FITC Apoptosis Detection Kit (YEASEN, Shanghai) was used to evaluate apoptosis. Briefly, cells were digested by 0.25% trypsin and centrifuged at 148 × g for 5 min at room temperature. Then cells were washed with PBS was used to wash and resuspended in 100 µl binding buffer. Afterward, cells were stained with incubated in darkness with 5 µl of Annexin V-FITC and propidium iodide (PI) for 15 min at room temperature. Flow cytometry (BD Biosciences, San Jose, USA) was used to calculate the apoptotic rates on FlowJo 10.07 software (FlowJo LLC).

### Quantitative reverse-transcription PCR (qRT-PCR)

PC12 cells and brain tissues were collected, and then RNA was extracted using TRIzol reagent (nitrogen source), isopropanol precipitation, and 75% ethanol washing. The resulting RNA pellet was dissolved in RNase-free water. cDNA and gRNA were reverse-transcribed from RNA using a transcription kit (TRAGEN). qRT-PCR was conducted using 20 ng of cDNA and the SYBR Green mixture (Shanghai Yisheng). U6 and GAPDH were used as the internal control for miRNA and mRNA, respectively.

### Western blotting

PC12 cells were placed in ice-cold lysis buffer for 30 min (Beyotime). The supernatant was collected via centrifugation at 1,120 × g for 20 min at 4°C. Protein concentration was calculated using a BCA kit (Pierce, USA). Then, the proteins were separated using 10% SDS-PAGE and transferred onto PVDF membranes, which were blocked with 5% nonfat milk. This was followed by overnight incubation at 4°C with the following primary antibodies (1:1000): anti-caspase3, anti-bcl-2, anti-bax, anti-PTEN (Boster Biological Technology, Wuhan, China). The proteins were then incubated with goat–anti-rabbit antibody (Abcam, Shanghai) and treated with BeyoECL Plus (Beyotime, Shanghai). Protein bands were visualized using a ECL kit (KeyGEN BioTECH, Nanjing, China).

### Luciferase reporter assay

The 3ʹ-UTRs of LOC366613 and PTEN, with wild-type and mutant (mut) binding sites for miR-532-5p, were amplified using PCR and cloned into pGL3 vectors (Promega, Madison, WI, USA). PC12 cells were co-transfected with the generated plasmids and either miR-532-5p or miR-NC, using Lipofectamine 2000 (Invitrogen). Luciferase activity was analyzed using the Dual-Luciferase System (Promega, Madison, WI, USA) following manufacturer protocols.

### RNA pull-down assay

An RNA pull-down assay was performed to confirm if LOC366613 and PTEN in PC12 cells could be pulled down by miR-532-5p. Biotin-labeled LOC366613 and PTEN were transcribed using Biotin RNA Labeling Mix and T7 RNA polymerase (Roche). After 2 d of transfection, PC12 cells were lysed and mixed with biotin-labeled LOC366613, PTEN, and streptavidin agarose magnetic beads at 4°C for 1 h. Western blotting was used to detect the protein levels of retrieved Ago2, and LOC366613 and PTEN expression was determined using qRT-PCR.

### Statistical analysis

Each independent experiment was performed in triplicate. All statistically analyzed were used SPSS17.0 software (SPSS, Inc.). Data are expressed as mean ± SD. One-way ANOVA was used to evaluate the differences among groups. The differences between the two groups were analyzed by student t-test. P < 0.05 was defined as significant.

## Results

### LOC366613

#### LOC366613 was upregulated in cerebral I/R–injured mice and PC12 cells

**Figure 1.**
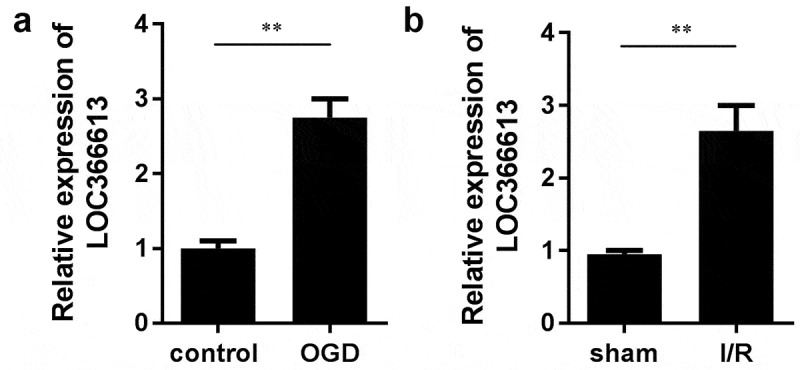
The expression of LOC366613 cerebral I/R injury in vivo and in vitro. (a)The expression of LOC366613 in PC12 cells treated with OGD treatment. (b)The expression of LOC366613 in MCAO mice. **P < 0.01

To investigate the role of LOC366613 in cerebral I/R injury, we established a mouse MCAO model and a PC12 cell model to detect the expression of LOC366613 both *in vivo* and *in vitro*. After OGD/R was induced, LOC366613 expression was upregulated in PC12 cells (Figure 1(a)). This was consistent with the findings of the *in vivo* assay, in which the expression of LOC366613 was significantly increased in I/R mice (Figure 1(b)).

#### *LOC366613 knockdown alleviated* in vivo *cerebral I/R injury*

**Figure 2.**
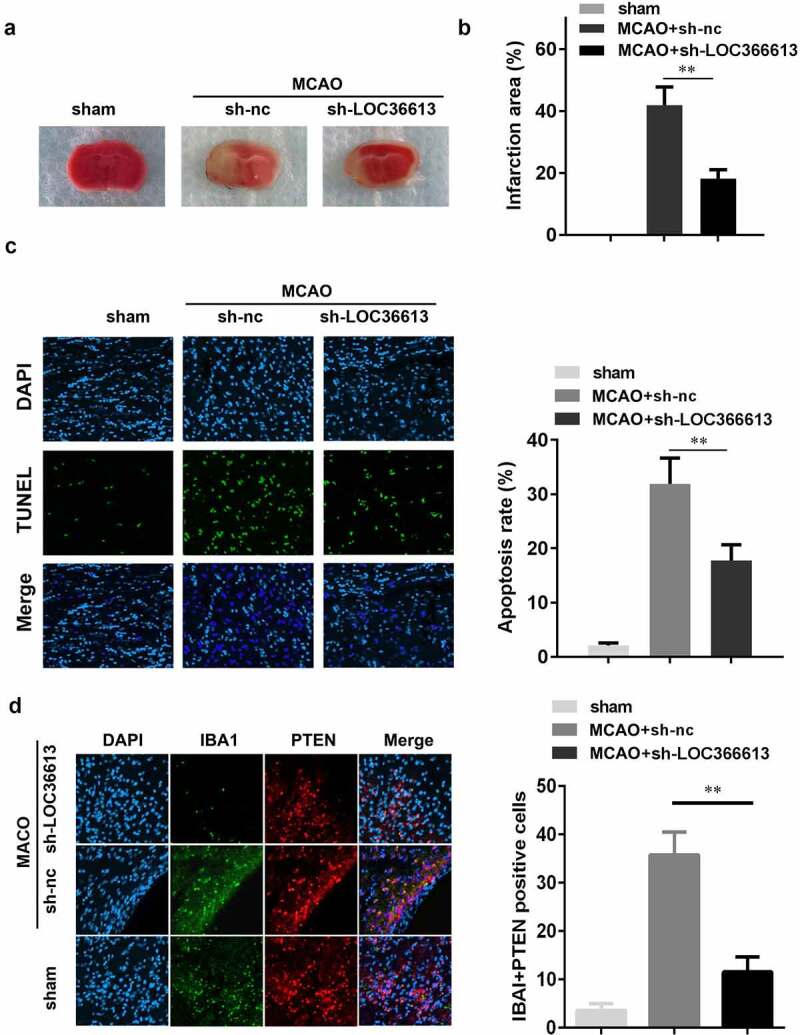
Knockdown of LOC366613 alleviated cerebral ischemia-reperfusion injury in mice. (a) Representative images of TTC staining of cerebral. (b) The area of CI. (c) The apoptosis of mice neuron determined by TUNEL staining. (d) Representative images of neurons visualized by fluorescence microscope. **P < 0.01

To further explore the role that LOC366613 plays in cerebral I/R injury, I/R mice were injected with sh-LOC366613 lentiviral vectors. This allowed us to assess the effect of LOC366613 silencing *in vivo*. TTC staining showed that Ad-sh-LOC366613 reduced the I/R injury–induced infarct area more than Ad-sh-NC did (Figure 2(a)). I/R surgery significantly increased the infarct volume in the experimental group compared with that in the control group (Figure 2(b)), whereas Ad-sh-LOC366613 reduced the infarct volume. TUNEL assay confirmed that knockdown of LOC366613 significantly decreased the amount of apoptosis in PC12 cells (Figure 2(c)). Moreover, knockdown of LOC366613 significantly decreased the number of IBA1+PTEN positive cells compared with MCAO+sh-NC group (Figure 2(d)).

#### LOC366613 knockdown alleviated damage in OGD-treated PC12 cells

**Figure 3.**
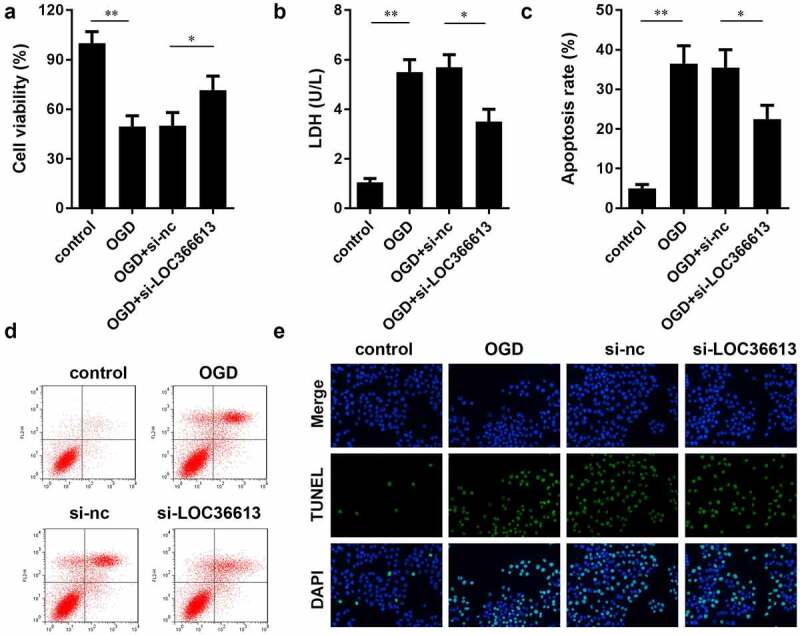
Knockdown LOC366613 attenuate I/R injury by OGD-induced. (a) The effect of LOC366613 silencing on PC12 cells viability. (b) The level of LDH. (c and d) The apoptosis of PC12 cells determined by flow cytometry. (e) The apoptosis of PC12 cells detected by TUNEL assay.*P < 0.05.**P < 0.01

To further explore the role of LOC366613 in cerebral I/R injury, we examined the effects of LOC366613 on cell proliferation, apoptosis, and LDH expression in OGD-induced PC12 cells. As shown in Figure 3(a-E), in the OGD-induced group, the LDH expression level and PC12 cell apoptosis rate were significantly increased compared to those in the control group, while PC12 cell viability was significantly decreased. In the OGD+si-LOC366613 group, the LDH expression level and PC12 cell apoptosis rate were decreased compared with those in the OGD-induced group, while PC12 cell viability was increased. BD results showed that OGD promoted the apoptosis of PC12 cells; the OGD-induced group experienced more cell deaths than the control group. Moreover, the rate of PC12 cell apoptosis was reduced in the OGD+si-LOC366613 group compared to that in the OGD-induced group. The TUNEL assay showed that knockdown of LOC366613 decreased OGD-induced PC12 cell apoptosis.

#### LOC366613 targeted miR-532-5p

**Figure 4.**
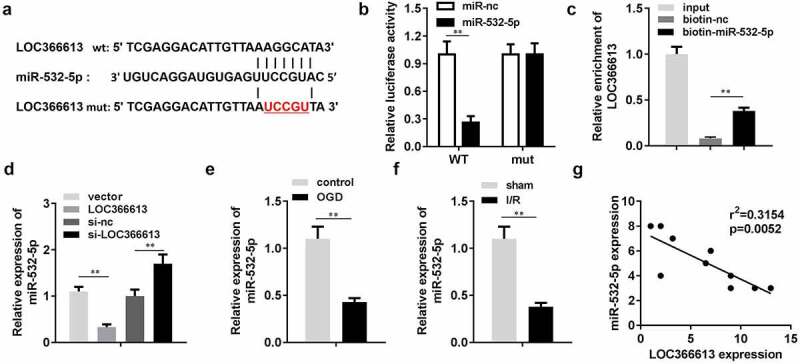
LOC366613 sponged miR-532-5p. (a) The binding sites between LOC366613 and miR-532-5p. (b) The binding sites verified by luciferase report assay. (c) The interaction between LOC366613 and miR-532-5p confirmed by RNA pull-down assay. (d) The expression of miR-532-5p. (e) The expression of miR-532-5p *in vitro*. (f)The expressions of miR-532-5p *in vivo*. (g) Correlation analysis the expression of miR-532-5p and LOC366613. **P < 0.01

lncRNAs modulate the expression of miRNAs by binding to their 3ʹ untranslated regions [11–13]. Therefore, we investigated the role of LOC366613 in miRNA suppression. According to the miRDB database, miR-532-5p was predicted to be a potential target of LOC366613 (Figure 4(a)). The luciferase assay showed that luciferase activity was significantly decreased by miR-532-5p in the pGL3-wt–LOC366613 co-transfection group (Figure 4(b)), while there were no significant changes in the pGL3-mut–LOC366613 co-transfection group. Additionally, the RNA pull-down assay verified interactions between miR-532-5p and LOC366613 (Figure 4(c)); miR-532-5p levels were significantly decreased by LOC366613, but increased by LOC366613 siRNA (Figure 4(d)). Next, we determined the expression levels of miR-532-5p, both *in vivo* and *in vitro*. After OGD, miR-532-5p expression was downregulated. This was consistent with the results of the *in vivo* assay; miR-532-5p expression in I/R mice was also reduced (Figure 4(e, F)). Correlation analysis showed that LOC366613 negatively regulated the expression of miR-532-5p (Figure 4(g)).

#### miR-532-5p reversed LOC366613-induced cerebral I/R injury

**Figure 5.**
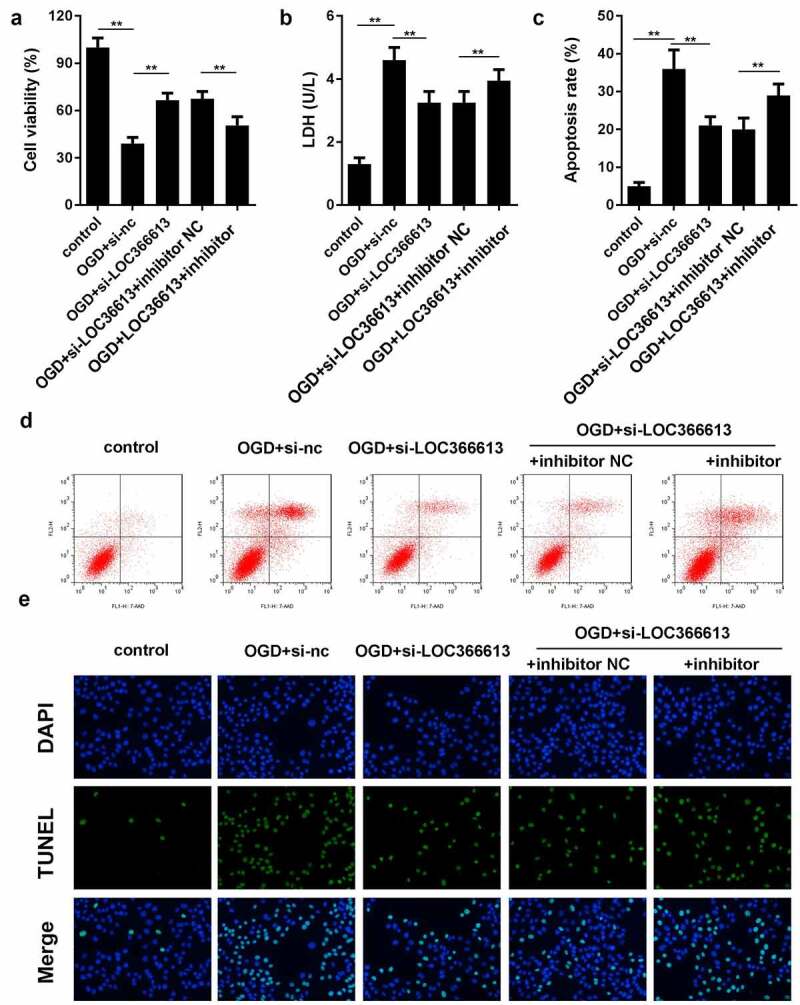
Downregulation of miR-532-5p reversed the effects of LOC366613 knockdown on PC12 cells. (a) Cell viability was detected by MTT assay. (b) The level of LDH in PC12 cells. (c and d) The apoptosis of PC12 cells determined by flow cytometry. (e) The apoptosis of PC12 cells detected by TUNEL assay. **P < 0.01

We performed a rescue experiment to further confirm the relationship between LOC366613 and miR-532-5p by co-transfecting si-LOC366613 and miR-532-5p inhibitors. As shown in Figure 5(a-C), the LDH expression level and PC12 cell apoptosis rate increased in the OGD+si-LOC366613+miR-532-5p inhibitor group compared to those in the OGD+si-LOC366613 group, while PC12 cell viability decreased. BD results indicated that PC12 cell apoptosis was promoted in the OGD+si-LOC366613+miR-532-5p inhibitor group compared with that in the OGD+si-LOC366613 group. The TUNEL assay showed that OGD+si-LOC366613–induced PC12 cell apoptosis was reversed by the addition of miR-532-5p inhibitors.

#### PTEN was a target of miR-532-5p

**Figure 6.**
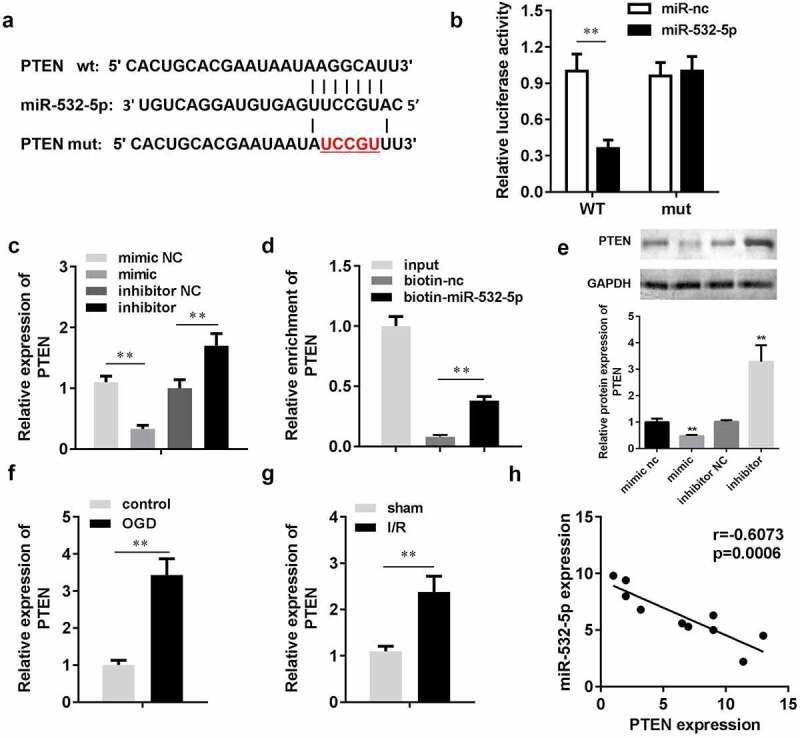
miR-532-5p negatively regulated PTEN. (a) The binding sites between miR-532-5p and PTEN. (b) The binding sites verified by luciferase report assay. (c) The expression of PTEN *in vitro* detected by qRT-PCR. (d) The interaction between PTEN and miR-532-5p confirmed by RNA pull-down assay. (e) The protein expression of PTEN *in vitro*. (f) The mRNA expression of PTEN *in vitro*. (g) The expressions of PTEN *in vivo*. (h) Correlation analysis the expression of miR-532-5p and PTEN. **P < 0.01

Targetscan (targetscan.org) predicted that *PTEN* was a target of miR-532-5p. The target binding region between miR-532-5p and *PTEN* is shown in Figure 6(a). The luciferase assay showed that miR-532-5p significantly decreased luciferase activity in the pGL3-wt-PTEN co-transfection group, while there were no significant changes in the pGL3-mut-PTEN co-transfection group. Moreover, *PTEN* expression levels were significantly decreased by miR-532-5p mimics, but increased by miR-534-5p inhibitors. The RNA pull-down assay also verified the interaction between *PTEN* and miR-532-5p. To further characterize the relationship between miR-532-5P and *PTEN*, we detected PTEN protein expression. Western blotting results showed that miR-532-5p mimics inhibited PTEN protein expression, while miR-532-5p inhibitors promoted it. In the *in vitro* assay, PTEN expression was upregulated after OGD. This was consistent with data gathered from the *in vivo* assay, in which PTEN expression was significantly increased in I/R mice. Correlation analysis showed that miR-532-5p negatively regulated PTEN expression.

## Discussion

Cerebral hypoxic-ischemic necrosis is considered a sign of CI exacerbation. Inhibiting inflammatory factor expression and neuronal cell apoptosis has been shown to be an effective method for treating cerebral I/R injury [28–30]. Therefore, exploring the molecular mechanism of cerebral I/R injury may provide new ideas for the development of more effective treatments for infarction. In this study, we established a mouse MCAO model and an *in vitro* OGD-induced PC12 cell line. Our results show that LOC366613 had a stimulating effect on the brain, promoting PC12 cell apoptosis and LDH expression. Interestingly, our results indicated that LOC366613 modulated the process of CI by regulating the miR-532-5p/PTEN pathway [31].

lncRNAs play crucial roles in cerebral I/R injury. Mutations and expression alterations might promote or inhibit cerebral I/R injury [32–34]. For instance, lncRNA MEG3 activates caspase1 signaling by targeting the miR-485/AIM2 axis and increasing pyroptosis, which in turn promotes cerebral I/R injury [35]. Downregulation of lncRNA Gm11974 alleviates neuronal cell apoptosis and significantly reduces cell death rates in rats with cerebral I/R injury [14]. Another study showed that *MIAT* overexpression reduces Neuro2A cell apoptosis through the miR-211/GDNF axis and relieves cerebral I/R injury in rats [36]. In our study, LOC366613 was upregulated in both cerebral I/R injury models and *in vitro* PC12 cell lines, with LOC366613 knockdown increasing the viability of PC12 cells and inhibiting cell apoptosis. The latter of these is a key player in the onset and development of cerebral I/R injury [5,6]. Therefore, knockdown of LOC366613 protects against cerebral I/R injury by inhibiting cell apoptosis and LDH expression. However, the underlying molecular mechanisms by which these processes occur remain unclear.

Numerous reports have confirmed that lncRNAs act as molecular sponges, regulating various biological functions and miRNA expression [37,38]. miRNAs play a key role in the onset and development of cerebral I/R injury, as well [22,39,40]. A study found that miR-124-5p overexpression significantly inhibits NF-κB signaling activation and the expression of various inflammatory factors by regulating NOX2 [41]. Another study found that miR-224-3p overexpression alleviates apoptosis in cerebral I/R injury by targeting FIP200 [42]. In our study, miR-532-5p was shown to be the target of LOC366613 and was downregulated in both the cerebral I/R injury model and PC12 cell line. Additionally, the miR-532-5p inhibitor partially reversed the effects of LOC366613 knockdown on cell viability, LDH release, and PC12 cell apoptosis. Moreover, previous studies have shown that *PTEN* is a direct downstream target of miR-532-5. Therefore, we propose that LOC366613 regulates PTEN expression via miR-532-5p.

Recent studies have confirmed that PTEN plays a key role in promoting cerebral I/R injury [27,43]. A previous study found that PTEN reverses the protective effect that miR-130a has on cerebral I/R injury [44]. Similarly, the results of our study indicate that *PTEN* is a target of miR-532-5p, which negatively regulates PTEN. Here, the miR-532-5p inhibitor reversed the increased cell viability, decreased LDH expression, and decreased the rate of cell apoptosis induced by LOC366613 knockdown. Therefore, we concluded that LOC366613 participates in cerebral I/R injury by inhibiting miR-532-5p expression.

## Conclusion

In this study, LOC366613 and PTEN were upregulated in a model of cerebral I/R injury, whereas miR-532-5p was downregulated. LOC366613 regulates the PTEN signaling pathway by acting as a sponge of miR-532-5p, aggravating cerebral I/R injuries. Therefore, the LOC366613/miR-532-5p/PTEN signaling cascade may be a potential target for the treatment of cerebral I/R injury.

## Data Availability

The datasets generated for this study are available on request to the corresponding author.

## Highlights

1. lncRNAs play crucial roles in CI injuries.

2. Knockdown of LOC366613 suppressed the injuries *in vivo* and *in vitro*.

3. LOC366613 suppressed the apoptosis of PC12 cells via regulating miR-532-5p/PTEN axis.
